# Band-Pass Filtering Cross-Polarization Converter Using Transmitarrays

**DOI:** 10.3390/ma14092109

**Published:** 2021-04-22

**Authors:** Jialin Feng, Hongyu Shi, Jianjia Yi, Anxue Zhang, Zhuo Xu

**Affiliations:** 1 Ministry of Education Key Laboratory for Multifunctional Materials and Structures, Xi’an Jiaotong University, Xi’an 710049, China; fenghui@stu.xjtu.edu.cn; 2 School of Information and Communications Engineering, Xi’an Jiaotong University, Xi’an 710049, China; anxuezhang@xjtu.edu.cn; 3 The Electronic Materials Research Laboratory, Key Laboratory of the Ministry of Education, Xi’an Jiaotong University, Xi’an 710049, China; xuzhuo@xjtu.edu.cn

**Keywords:** band-pass filtering, cross-polarization converter, transmitarray

## Abstract

Microwave devices with polarization conversion and band-pass filtering response have great application prospects on radomes. Here, the concepts of band-pass filters and cross-polarization converters are combined to realize a band-pass filtering cross-polarization converter with an extremely high polarization-conversion ratio. Most importantly, the device has an excellent out-of-band rejection level, above 30 and 40 dB for the lower and upper edges, respectively. In addition, the transmission zeros of the passband can be flexibly tuned independently. The band-pass filtering polarization converter was simulated, fabricated, and measured, and the measured results were found to be in good agreement with the simulation results.

## 1. Introduction

Polarization is one of the basic properties of electromagnetic (EM) waves, and EM polarization manipulation is widely used to design antennas and radomes; thus, polarization converters have been extensively investigated [1,2,3]. In recent years, metasurfaces have been widely employed in polarization converters, since they are thinner and have improved performances compared with traditional wave plates based on natural materials and anisotropic material [4]. Reflective metasurface polarization converters are usually dual-band or multi-band, broadband, and highly efficient [5,6]. Transmissive polarization converters are required in some applications to avoid the interference between the reflected and incident waves. They are usually obtained via multilayer meta-structures, leading to a complex design process and a simple function [7,8]. Recently, a simpler design method for transmissive polarization converter was proposed using coupling-propagation-decoupling structures [9]. Through this method, multifunctional polarization converters with beam redirection or vortex beam generation were obtained [10,11,12]. These beam reshape functions base on the phase tuning. However, phase tuning cannot be applied to achieve frequency selective functions.

Frequency selective surfaces (FSSs) have a filtering response and have been widely used in antenna radomes [13,14,15,16,17,18,19]. However, most FSS designs have an out-of-band rejection below 20 dB at the lower and upper edges of the passband, which can hardly satisfy the requirements of recent radar systems. In addition, few FSSs can simultaneously achieve frequency selection and polarization conversion. A band-pass frequency-selective polarization converter was proposed using anisotropy structures, but the obtained roll-off frequency response is poor [20]. Another FSS with polarization conversion was designed based on a substrate-integrated waveguide (SIW) [21]; however, it exhibits only one transmission zero in the lower stopband, leaving a low out-of-band rejection at higher stopband. Therefore, multifunctional FSSs with polarization conversion, a sharp roll-off frequency response, and a superior out-of-band rejection in both the lower and upper stopbands are still required.

In this work, a band-pass filtering cross-polarization converter with an enhanced frequency selectivity and a high polarization-conversion ratio was realized. Inspired by aperture-coupled transmitarrays, the designed band-pass filtering cross-polarization converter can operate from 5.87 to 6.13 GHz with a cross-polarized transmittance higher than −1 dB and a co-polarized transmittance lower than −55 dB. Furthermore, transmission zeros were introduced in both the lower and upper stopbands at 5.72 and 6.72 GHz, respectively. Notably, the band-pass filtering response exhibits an excellent out-of-band rejection, over 30 and 40 dB for the lower and upper stopbands, respectively. Moreover, the positions of the transmission zeros at the lower and upper stopbands can be independently tuned, which makes it a fixable design for different applications. To confirm the proposed method, the designed band-pass filtering cross-polarization converter was simulated, fabricated, and measured.

## 2. Band-Pass Filtering Cross-Polarization Converter Design

**Figure 1:**
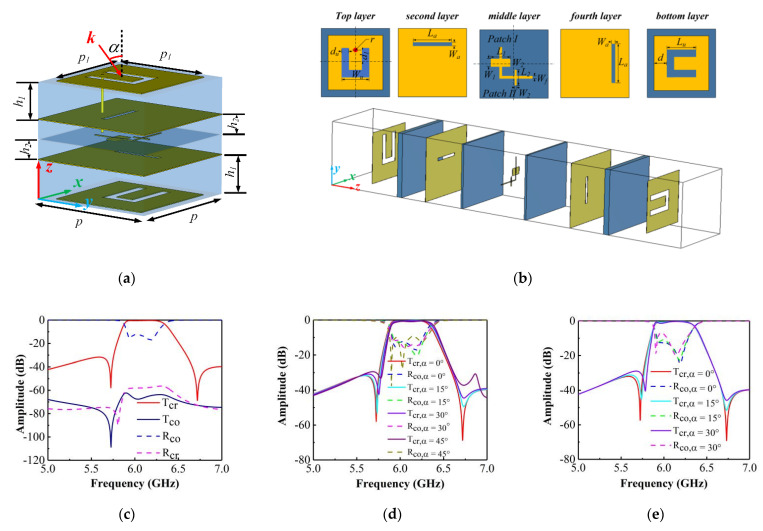
(**a**) Configuration of the proposed band-pass filtering cross-polarization converter unit cell; (**b**) five layers of copper sheets; (**c**) simulated T_cr_, R_co_, T_co_ and R_cr_ of the unit cell. Simulated T_cr_ and R_co_ of the unit cell under oblique incidence in the (**d**) transverse electric (TE) modes and (**e**) and transverse magnetic (TM) modes.

The designed band-pass filtering cross-polarization converter is composed of four layers of dielectric and five layers of metal patterns, as shown in Figure 1a. The dielectric substrate is Taconic TLY-5, which is characterized by a relative dielectric constant of 2.2 and a loss tangent of 0.0009. The structure of the proposed band-pass filtering cross-polarization converter unit cell is depicted in Figure 1b. Here, the blue and yellow regions denote the dielectric substrate and the copper sheet, respectively. The values of the geometric parameters of the design are listed in Table 1.

**Figure 2:**
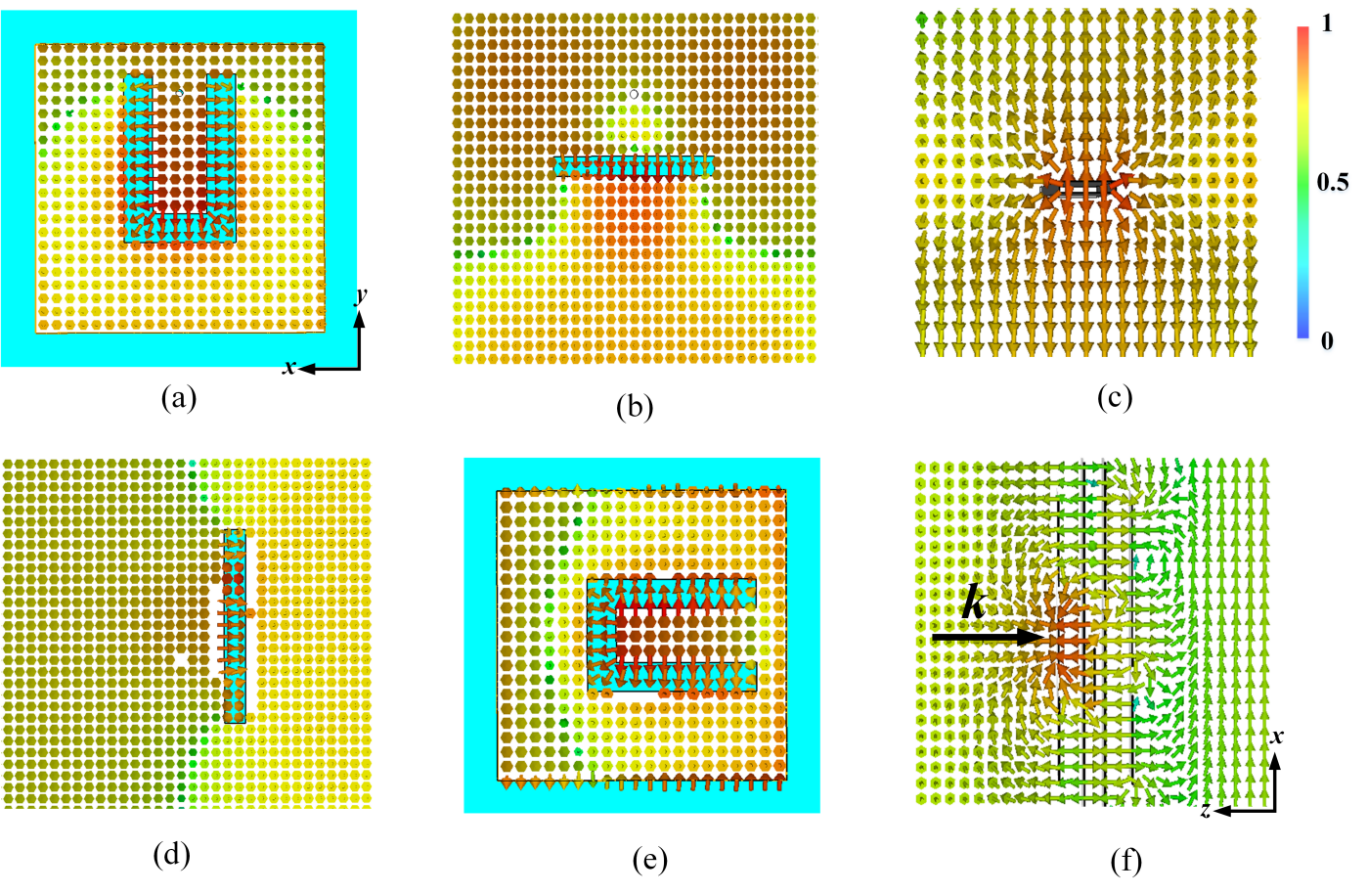
Unit cell at 6.1 GHz: (**a**) E-field distribution in the top layer, (**b**) E-field distribution in the second layer, (**c**) E-field distribution around the metallic line, (**d**) E-field distribution in the fourth layer, (**e**) E-field distribution in the bottom layer, and (**f**) E-field distribution in the unit cell.

The proposed design has a relatively high efficiency since only the top and bottom layers resonate, thus significantly reducing the insert loss caused by resonances. The top and bottom layers are patches which couple and decouple the incident EM waves, respectively. The bottom layer of the unit cell can be obtained from the top layer via a rotation of 90°. Notably, in contrast to the bottom layer, a shorting pin is embedded on the top layer and passes through the top two layers of the dielectric substrates. This shorting pin introduces a transmission zero at the lower stopband. These patches can be regarded as slot-coupled patch antennas [22] with a U-slot and decide the overall working frequency. The coupling slots are located on the ground layer (the second and fourth metallic layers). Figure 2 shows the E-field distribution at 6.1 GHz, when y-polarized incident EM waves propagating along z-axis hit the unit cell, the patch in top layer resonates as shown in Figure 2a and then couples the incoming EM waves to the metallic line on the middle layer through the coupling slot. Figure 2b show the E-field distribution in the second layer, it can be seen that the E-field near the coupling slot is strong and has a tangential component. The metallic line, constructed of an L-shaped stripline and two patches with different dimensions, can be consider as a filter structure based on stepped-impedance resonators (SIRs) [23,24]. This SIR filter structure in the middle layer results in a transmission zero at the upper stopband of the passband. The structure in the middle layer can transform the wave propagation along the y-axis into a wave propagation along the x-axis. The wave propagation along the x-axis is then coupled to the bottom layer through the coupling slot on the fourth layer since the tangential E-field excites the propagation mode in the stripline, as shown in Figure 2c. Figure 2d show the E-field in the fourth layer, we can observe that the E-field around the coupling slot has a tangential component. Eventually, the bottom layer decouples the wave into a space wave propagation with a crossed polarization as shown in Figure 2f. Furthermore, through the procedure of coupling-propagation-decoupling used in this design, an excellent band-pass filtering response is achieved via the two transmission zeros, which will be discussed later.

The simulation results are shown in Figure 1c–e, where T represents the amplitude of the transmission, and R denotes the reflection. The subscripts cr and co stand for cross- and co-polarized, respectively. Figure 1c shows the simulation results for normal incidence. It can be seen that the proposed design can achieve transmissive cross-polarization conversion and band-pass filtering in the range of 5.87–6.13 GHz; additionally, T_cr_ is close to 0.94, and R_co_ is below −12 dB in the operating band. At the same time, the co-polarized transmittance T_co_ and cross-polarized reflectance R_cr_ are both below −55 dB, approximating to zero, which ensures an extremely high polarization purity of the transmitted wave. The transmission zeros of the lower and upper stopbands are located at 5.72 and 6.73 GHz, respectively. Notably, the out-of-band rejection of the lower and upper stopbands of the passband are higher than 30 and 40 dB.

In addition, the properties of the proposed band-pass polarization converter were explored under different incident angles in the transverse electric (TE) and transverse magnetic (TM) modes. The simulated T_cr_ and R_co_ are shown in Figure 1d,e, respectively, where α  denotes the angle of incidence. Figure 1d depicts T_cr_ and R_co_ in the TE mode. When the α value increases up to 45°, T_cr_ is 0.9 and R_co_ slowly rises to −10 dB; thus, the proposed band-pass polarization converter has still a good filtering capacity. The simulated T_cr_ and R_co_ in the TM mode are shown in Figure 1e. In this case, it can be observed that the proposed band-pass polarization converter can retain a good filtering response only for incident angles below 30°.

## 3. Band-Pass Filtering Mechanism and Discuss

The filtering mechanism of the proposed device is explained by comparing it with two other structures, namely (I) the designed unit cell without the SIR filter structure and (II) the unit cell without the shorting pin.

**Figure 3:**
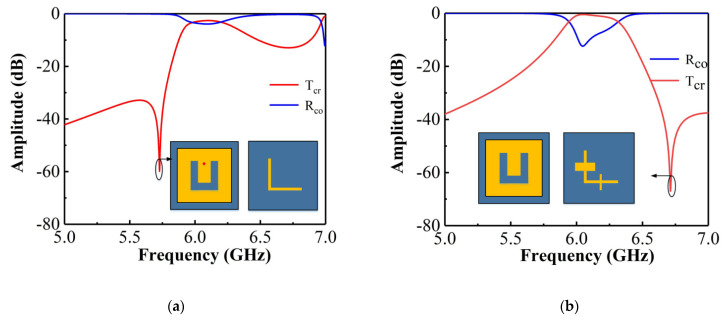
(**a**) T_cr_ and R_co_ simulated for case (I); (**b**) T_cr_ and R_co_ simulated for case (II).

Figure 3 shows the simulated T_cr_ and R_co_ in cases (I) and (II). As shown in Figure 3a, a transmission zero exists in the lower stopband of the passband; however, no transmission zeros are visible in the upper stopband. Additionally, T_cr_ is 0.74 resulting from a higher R_co_ value of 0.64. Thus, the shorting pin leads to the transmission zero being in the lower stopband of the passband. Furthermore, the filter structure on the middle layer influences both the impedance matching and the transmission zero in the upper stopband, as will be discussed later. Regarding case (II), the model has the same dimensions as the final design without the shorting pin. The introduced SIR filtering structure can be adjusted to achieve optimized filtering response and impedance matching. The simulated transmission response of the model in case (II) is shown in Figure 3b. It can be seen that the impedance matching is improved, and a transmission zero is introduced in the upper stopband of the passband. Thus, combining cases (I) and (II) results in a superior frequency selective performance.

**Figure 4:**
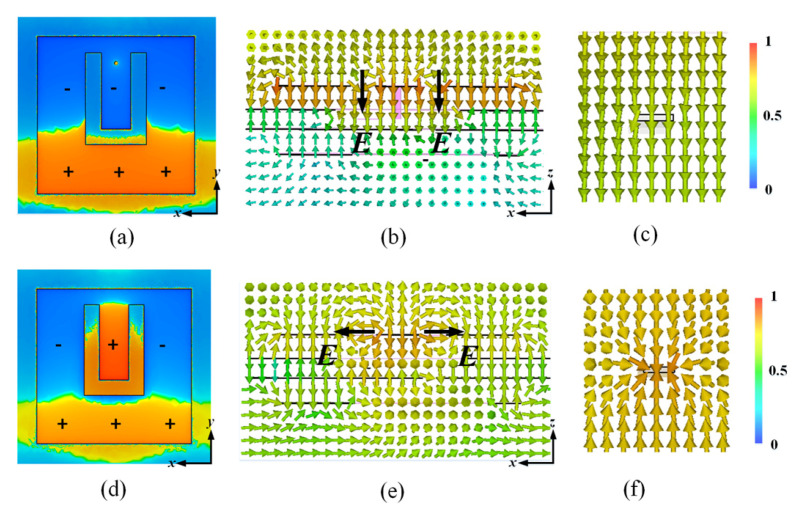
Unit cell with shorting pin at 5.72 GHz: (**a**) E-field distribution on the top patch, (**b**) E-field distribution in the unit cell, and (**c**) E-field distribution around the metallic line. Unit cell without the shorting pin at 5.72 GHz: (**d**) E-field distribution in the top patch, (**e**) E-field distribution in the unit cell, and (**f**) E-field distribution around the metallic line.

In order to clarify the mechanism for generating the transmission zero in the lower stopband, Figure 4 shows the E-field distribution on the U-slot patch at 5.72 GHz for the unit cell with and without the shorting pin. As shown in Figure 4a, the resonance mode of the U-slot patch with the shorting pin is similar to that of a dipole. The EM waves cannot couple to the metallic line through the coupling slot, as the E-field near the stripline is always along the normal direction and ignores the stripline, as shown in Figure 4b,c. As illustrated in Figure 4d, the E-field distribution of the U-slot patch without the shorting pin is multipole. This results in a tangential E-field in the U-slot which excites the propagation mode in the stripline, as shown in Figure 4e,f. Therefore, EM waves can be first coupled into the stripline, and then be decoupled from the bottom U-slot patch leading to an undesired transmission. According to the above analysis, the transmission zeros in the lower and upper stopbands are caused by the shorting pin and the SIR filtering structure, respectively.

## 4. Discussion

**Figure 5:**
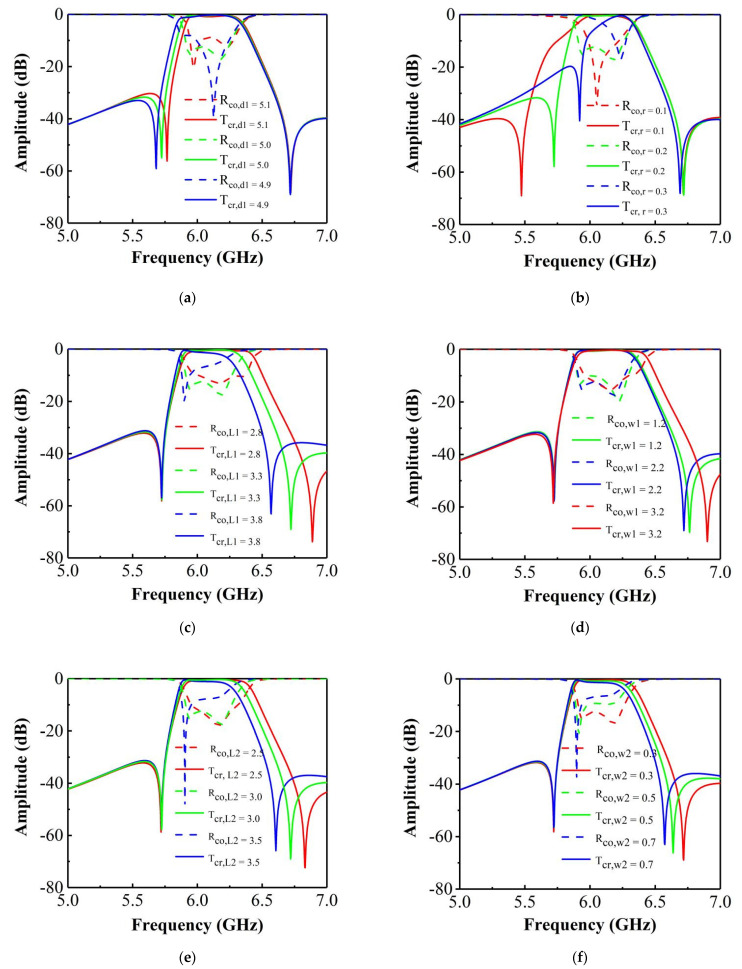
Effect of the shorting pin: (**a**) distance between the shorting pin and the center of patch *d*_1_; (**b**) radius of the shorting pin *r*; (**c**) length of Patch I, *L*_1_; (**d**) width of Patch I, *W*_1_; (**e**) length of Patch II, *L*_2_; (**f**) width of Patch II, *W*_2_.

To demonstrate the independent tunability property of the transmission zero positions in the lower and upper stopbands, a parametric study for the proposed design was carried out. It is believed that this study will also be beneficial for design of different applications using the proposed method. The results are shown in Figure 5, where the colored solid line denotes T_cr_, while the dashed line represents R_co_.

The effect of the shorting pin was investigated first. As shown in Figure 5a, the change of *d*_1_ has a slight effect on both the resonant frequencies and the transmission zero at lower frequency. When *d*_1_ increases from 4.9 to 5.1 mm, the transmission zero moves towards a higher frequency, and a declined out-of-band rejection is observed in the lower stopband in the passband. Furthermore, the sharp roll-off frequency response is retained during this process. From Figure 5b, it can be observed that the resonance frequencies and the transmission zero in the lower stopbands change clearly upon varying *r*: when the shorting pin has a bigger radius, the sharp roll-off rate in the lower stopband improves significantly; however, the out-of-band rejection quickly drops to 20 dB. Meanwhile, the transmission zero in the lower stopband moves towards a higher frequency and causes a narrower band.

The effect of the SIR filter structure geometry was then investigated. As shown in Figure 5c, when *L*_1_ varies from 2.8 to 3.8 mm, the transmission zero in the upper stopband moves towards a lower frequency, and the roll-off rate remains unchanged. However, the out-of-band rejection level drops with a bigger *L*_1_. In addition, as *L*_1_ varies, the roll-off rate and the position of the transmission zero in the lower stopband remain fixed. *L*_1_ also has a influence on the impedance matching. The effect of *W_1_*, was also studied. From Figure 5d, variations of *W_1_* only cause a change of the transmission zero at the upper stopband and of the resonance frequency for higher frequencies. A lager *W_1_* results in the transmission zero shifting towards a higher frequency. Notably, in contrast to varying *L*_1_, varying *W_1_* has little impact on the impedance matching. With reference to Figure 5e,f, it can be observed that *L_2_* and *W*_2_ have similar effects with *L*_1_. Therefore, an independent adjustment of the transmission zeros in the lower and upper stopbands can be achieved through optimizing the shorting pin and the SIR filter structure, respectively.

## 5. Measurement Results

**Figure 6:**
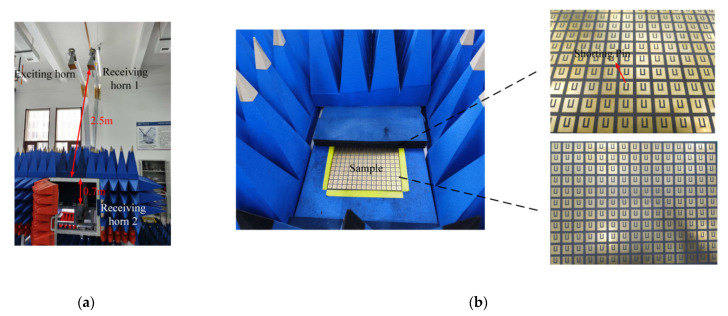

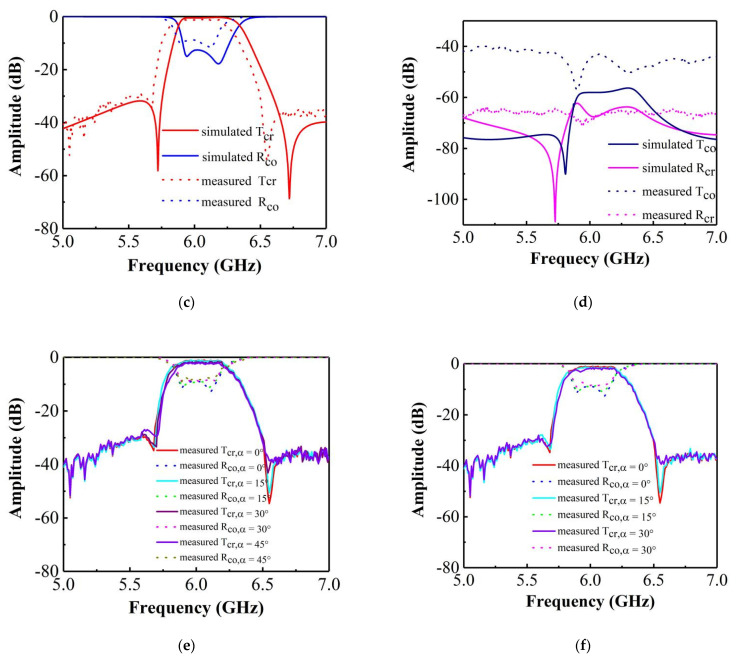
(**a**) The measurement setup; (**b**) front-view and back-view of sample; (**c**) the measured T_cr_, R_co_ for α = 0°; (**d**) the measured T_co_, R_cr_ for α=0°. Measured T_cr_, R_co_ under different incident angles in the (**e**) TE modes and (**f**) TM modes.

To confirm the viability of the proposed design, the band-pass filtering cross-polarization converter with a high conversion ratio was fabricated via the Printed Circuit Board (PCB) process. Due to the limitation of the size of the dielectric substrate, the dimension of the sample was of 220 × 300 mm^2^, and it contained 11 × 15 unit cells. Figure 6a shows the measurement setup, while Figure 6b show the front- and back-view of the fabricated sample.

The transmittance and the reflectance were measured using a vector network analyzer (Agilent E8363b). Three horn antennas were used for exciting and receiving antennas. The fabricated sample was placed on a platform and surrounded by the absorbers. When measuring R_cr_ and R_co_ of the proposed band-pass filtering cross-polarization converter, the two horn antennas above the sample were used as exciting and receiving antenna. The distance between these two horn antennas and sample is 2.5 m. The horn antenna located under the platform served as the receiving antenna during the measurement process of T_cr_ and T_co_, and the distance from this antenna to the sample is 0.7 m. The measured results are shown in Figure 6c,d for α=0°. The measured transmission zeros of the lower and upper stopbands are located at 5.69 and 6.52 GHz, respectively. The measured transmittance of the cross-polarization is −1 dB. The transmission zero of the upper edge moves towards a lower frequency due to the machining error on the SIR filter structure. This results in a narrower operating band and a lower cross-polarization transmittance. From Figure 6d, it can be seen that both the measured T_co_ and R_cr_ are below −40 dB; the measured and simulated T_co_ and R_cr_ are not very consistent due to insufficient dynamic range of the experimental equipment and the machining error. However, the measured T_co_ and R_cr_ still retain an extremely low level, approximately zero, indicating that a high efficiency of the cross-polarization conversion can be ensured. Figure 6e,f show the measured T_co_ and R_cr_ under different incident angles in the TE and TM modes, respectively. Thus, it can be concluded that the fabricated band-pass filtering cross-polarization converter exhibits a high polarization-conversion ratio and an excellent band-pass filtering response.

## 6. Conclusions

In conclusion, a band-pass filtering cross-polarization converter with a high polarization conversion ratio was designed, fabricated, and measured. The design was inspired by transmitarrays with a multi-layer structure. A cross-polarization conversion and a band-pass filtering from 5.87 to 6.13 GHz could be achieved, as well as a cross-polarized transmittance over 0.94 and a co-polarized reflectance below −12 dB. On the other hand, the cross-polarized reflectance and the co-polarized transmittance were always found to be below −55 dB. Thus, the polarization purity of the transmitted wave could be ensured. The transmission zeros were found to be located at 5.72 and 6.73 GHz. In addition, by varying the size and position of the shorting pin and the SIRs, the two transmission zeros could be tuned independently.

## Figures and Tables

**Table 1:** Geometric parameters of the unit cell.

Parameter	*C_1_*	*C_2_*	*d*	*d_u_*	*d*_1_	*h_1_*	*h_2_*	*L_a_*	*L_f_*	*L*_1_
**Value (mm)**	0.7	0.2	5.0	1.6	5.0	1.524	0.508	8.2	7.1	3.3
**Parameter**	*L_u_*	*L*_1_	*L_2_*	*P*	*P_1_*	*r*	*W_f_*	*W_u_*	*W_1_*	*W*_2_
**Value (mm)**	9.35	3.3	3.0	20	16	0.2	0.2	0.8	2.2	0.3

## Data Availability

The data that support the findings of this study are available within the article.
